# Properties-Adjustable Alumina-Zirconia Nanolaminate Dielectric Fabricated by Spin-Coating

**DOI:** 10.3390/nano7120419

**Published:** 2017-11-29

**Authors:** Junbiao Peng, Jinglin Wei, Zhennan Zhu, Honglong Ning, Wei Cai, Kuankuan Lu, Rihui Yao, Hong Tao, Yanqiong Zheng, Xubing Lu

**Affiliations:** 1 School of Electronic and Information Engineering, South China University of Technology, Guangzhou 510640, China; psjbpeng@scut.edu.cn (J.P.); magicwei@foxmail.com (J.W.); 2 Institute of Polymer Optoelectronic Materials and Devices, State Key Laboratory of Luminescent Materials and Devices, South China University of Technology, Guangzhou 510640, China; zhu.zhennan@mail.scut.edu.cn (Z.Z.); c.w01@mail.scut.edu.cn (W.C.); kk-lu@foxmail.com (K.L.); tao.h@scut.edu.cn (H.T.); 3 Key Laboratory of Advanced Display and System Applications of Ministry of Education, Shanghai University, Shanghai 200072, China; zhengyq21cn@126.com; 4 Institute for Advanced Materials and Guangdong Provincial Key Laboratory of Quantum Engineering and Quantum Materials, South China Normal University, Guangzhou 510006, China; luxubing@scnu.edu.cn

**Keywords:** properties-adjustable, Al_2_O_3_-ZrO_2_ nanolaminate dielectric, spin-coating

## Abstract

In this paper, an alumina-zirconia (Al_2_O_3_-ZrO_2_) nanolaminate dielectric was fabricated by spin-coating and the performance was investigated. It was found that the properties of the dielectric can be adjusted by changing the content of Al_2_O_3_/ZrO_2_ in nanolaminates: when the content of Al_2_O_3_ was higher than 50%, the properties of nanolaminates, such as the optical energy gap, dielectric strength (V_ds_), capacitance density, and relative permittivity were relatively stable, while the change of these properties became larger when the content of Al_2_O_3_ was less than 50%. With the content of ZrO_2_ varying from 50% to 100%, the variation of these properties was up to 0.482 eV, 2.12 MV/cm, 135.35 nF/cm^2^, and 11.64, respectively. Furthermore, it was demonstrated that the dielectric strength of nanolaminates were influenced significantly by the number (n) of bilayers. Every increment of one Al_2_O_3_-ZrO_2_ bilayer will enhance the dielectric strength by around 0.39 MV/cm (V_ds_ ≈ 0.86 + 0.39n). This could be contributed to the amorphous alumina which interrupted the grain boundaries of zirconia.

## 1. Introduction

Oxide thin film transistors (TFTs) have attracted considerable interest because of their high-performance. In addition to oxide semiconductors, which were mostly investigated in the last few decades, the metal oxide dielectric plays an important role in the performance of TFTs. In recent years, as a novel structure of dielectrics, a nanolaminate dielectric has been studied and applied widely in optoelectronic devices [1,2]. Waggoner et al. found that nanolaminates can take advantage of different components thereby enhancing the performance and stability of the devices compared to single-layer dielectrics [2]. López et al. demonstrated that the optical properties of dielectrics can be modulated by varying the nanolaminate’s thickness [3]. The TiO*_x_* and AlO*_x_* nanolaminate dielectric has been applied in organic devices as moisture barriers by Nehm et al., and it is helpful to delay the degradation of OLEDs which was caused by moisture erosion [4]. Meanwhile, so many techniques have been developed to fabricate nanolaminate dielectrics, such as atomic layer deposition (ALD) [5], pulsed plasma-enhanced chemical vapor deposition [6], and cyclic chemical vapor deposition (CVD) [7]. However, the application of solution-process to fabricate nanolaminates were rarely reported, especially for spin-coating. Spin-coating has attracted more and more attention for low cost, simplicity, and high throughput [8]. Metal oxide dielectrics, such as Al_2_O_3_, HfO_2_, and ZrO_2_ can be obtained easily by spin-coating [9,10,11]. Meanwhile, it has the potential to realize fully transparent, flexible, and portable electronics [12,13].

In this work, we fabricated a series of nanolaminate dielectrics by spin-coating and the relevant structures and properties were investigated. Nanolaminate dielectric was prepared in two approaches: (1) changing the content of Al_2_O_3_ in nanolaminates with constant number of bilayers; and (2) changing the number of bilayers with a constant content of Al_2_O_3_. The results showed that the properties of the dielectric can be adjusted by controlling the content of the components in nanolaminates and the number of bilayers. It was demonstrated that the performance of nanolaminates fabricated by vacuum-process can also be realized in the solution process. Compared with the vacuum process, the nanolaminate dielectric fabricated by spin-coating succeeded in avoiding rigorous experimental conditions (such as a high ambient vacuum) and complex operation (such as controlling the deposition speed of the thin film) [2,14]. This paper provided a new idea to fabricate dielectrics by the solution process.

## 2. Materials and Methods

The precursor solution was prepared by dissolving ZrOCl_2_·8H_2_O and Al(NO_3_)_3_·9H_2_O in methoxyethanol, respectively. Substrates were cleaned by isopropanol, tetrahydrofuran, lotion, deionized water (twice), and isopropanol sequentially, and then dried at 80 °C in an oven. To activate the surface, substrates were treated by O_2_ atmospheric pressure plasma for 10 min [15]. The precursor was coated at 4000 rpm on substrate for 40 s and the resulting film was annealed in air for 1 h.

To find out the optimized annealing temperature for nanolaminate fabrication, ZrO_2_ and Al_2_O_3_ single layers were prepared. The resulting films were measured by X-ray reflectivity (XRR) to explore the relationship between thickness (as well as density) and annealing temperature. Then, the relationship between thickness and precursor concentration was also explored after the optimized temperature was confirmed. Finally, the results were analyzed by mathematical statistics and tested by Student’s *t*-test [16,17].

**Figure 1:**
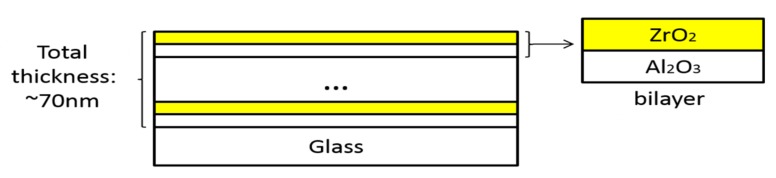
Plot of nanolaminates and bilayers on glass.

Al_2_O_3_-ZrO_2_ nanolaminates were fabricated by coating the precursor on substrate repeatedly, the resulting film will be annealed at optimized temperature for one hour in air before the next coating. The cross-sectional image of the nanolaminates is shown in Figure 1. The content of the components in the nanolaminates were controlled by the thickness ratio of Al_2_O_3_ (T_1_) and ZrO_2_ (T_2_). For example, if T_1_/T_2_ was 1:1 in bilayers, the content of Al_2_O_3_ was 50%. In this work, the content of Al_2_O_3_ was set to 0%, 25%, 50%, 75%, and 100%, and the number of bilayers was set to 1, 2, 3, and 6.

**Figure 2:**
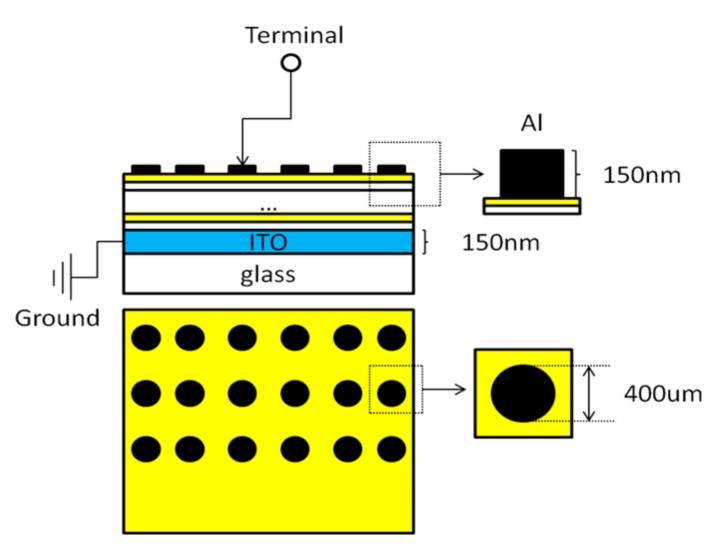
Plot of the MIM device.

Crystallizations of the nanolaminate dielectric was identified by X-ray diffraction (XRD) at 40 kV and 40 mA, with Cu-kα used as X-ray source. The scanning was carried out at the rate of 0.1°/s from 20° to 70°. A UV-VIS spectrophotometer was used to characterize the transmittance of nanolaminates in a range from 200 nm to 800 nm and E_g_ can be obtained by the Tauc formula [18]. The microstructure of nanolaminates was analyzed by HR-TEM and EDS. The crystallite size of nanolaminates was calculated by Scherrer’s equation [19]:

(1) D=kγ/Bcosθ

where D is the crystallite size, γ is the wavelength of X-ray (15.4056 nm), B is the full width at half maximum (FWHM), θ is the Bragg angle and k is the Scherrer constant (0.89). Substrates deposited with 150 nm indium tin oxide (ITO) were used to fabricate metal insulator metal (MIM) devices, as shown in Figure 2. ITO acts as the bottom electrode and top electrodes are prepared with aluminum (Al) deposited by an Edward Auto 500 unit. The diameter and thickness of Al electrodes are 400 μm and 150 nm, respectively. A semiconductor parameter analyzer was used to measure breakdown voltage under the direct current, the test voltage was applied from 0 V to 20 V with the step of 0.2 V. V_ds_ was the value of breakdown voltage divided by electrode separation distance. Capacitance was measured by a Keithley4200-SCS unit. Meanwhile, relative permittivity ($\varepsilon_{r}$) also can be calculated by the following formula [20]:

(2) $$\varepsilon_{r}=\;\frac{C\cdot d}{\varepsilon_{0}\cdot S}\;=\;\frac{C\cdot d}{\varepsilon_{0}\cdot \pi \cdot r^{2}}$$

where C, d, and S are the capacitance, thickness, and area of nanolaminates, respectively, and $\varepsilon_{0}$ = 8.854 × 10^−12^ F/m (permittivity in free space).

## 3. Results and Discussion

### 3.1. Optimized Annealing Temperature Exploration and Verification

**Figure 3:**
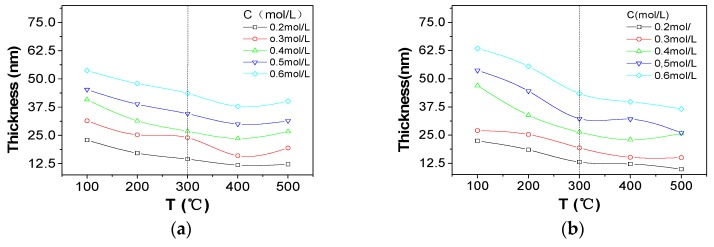

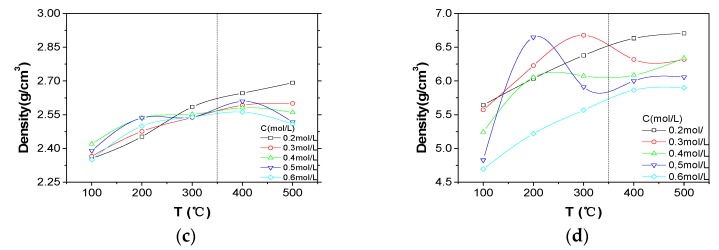
Thickness and density of Al_2_O_3_ and ZrO_2_ single layers tested by XRR: (**a**) the plot of thickness versus temperature (T) of Al_2_O_3_ single layers; (**b**) the plot of thickness versus temperature (T) of ZrO_2_ single layers; (**c**) the plot of density versus temperature (T) of Al_2_O_3_ single layers; (**d**) the plot of density versus temperature of ZrO_2_ single layers. C presented in the legend is the concentration of the solution.

To avoid the difference of properties caused by thickness and density, the optimized annealing temperature was explored by Al_2_O_3_ and ZrO_2_ single layers. As shown in Figure 3a,b, the variations of thickness of Al_2_O_3_ and ZrO_2_ single layers tended to be stable when the annealing temperature was above 300 °C. Similarly, the density fluctuation of ZrO_2_ and Al_2_O_3_ was also suppressed significantly when the temperature was higher than 350 °C, as shown in Figure 3c,d. The results showed that thickness and density were relatively stable when the annealing temperature was over 350 °C. Therefore, 400 °C (between 350 °C and 500 °C) was selected as the annealing temperature.

**Figure 4:**
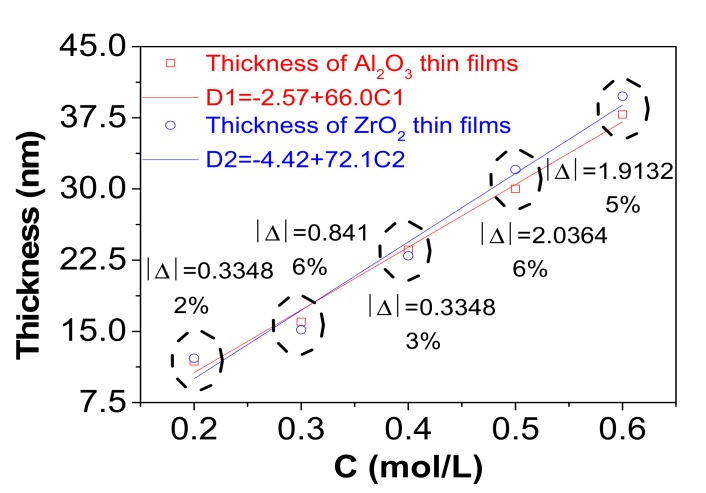
Plot of thickness versus concentration (C) of Al_2_O_3_ and ZrO_2_ single layers (annealed at 400 °C). |Δ| is the absolute difference value of thickness between Al_2_O_3_ and ZrO_2_. Percentage under the |Δ| is the relative error calculated by the ratio of |Δ| and thickness, the thickness is picked out from the smaller of Al_2_O_3_ and ZrO_2_. D1 and D2 are equations of linear regression of the thickness and precursor concentration.

Meanwhile, the percentage marked in Figure 4 showed little difference between the thickness of Al_2_O_3_ and ZrO_2_ single layers, which were coated by precursors with the same concentration. The result was analyzed by mathematical statistics theory, as shown by the linear regression equations in Figure 4. With the concentration increased by 0.1 mol/L, the thicknesses of Al_2_O_3_ and ZrO_2_ were increased by 6.6 nm and 7.21 nm respectively. The result was tested by *t*-test (α = 0.05). It was demonstrated that the result was reasonable since the test statistics of Al_2_O_3_ ($t_{{\mathrm{Al}}_{2}O_{3}}$ = 17.0264) and ZrO_2_ ($t_{{\mathrm{ZrO}}_{2}}$ = 11.3591) were higher than the critical value ($t_{1-\frac{\alpha }{2}}\left(n-2\right)=3.1824$). Thus, it is believed that the thickness of the layers which were fabricated by precursors with the same concentration were similar.

**Figure 5:**
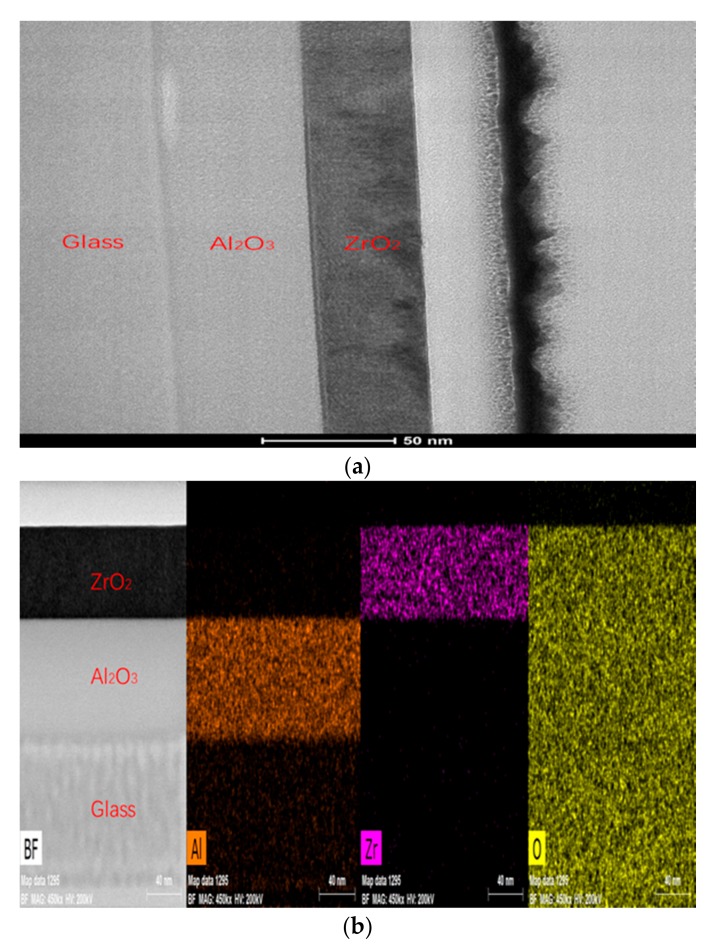
(**a**) The cross-sectional HR-TEM image of nanolaminates which contains one bilayer (the main parameters are shown in Table 4). (**b**) The cross-sectional EDS image of nanolaminates.

**Figure 6:**
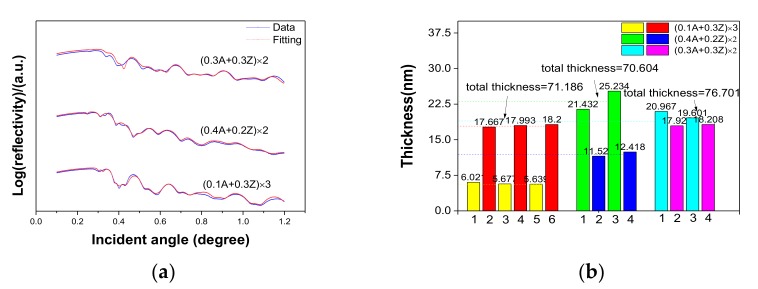
(**a**) The XRR measurements of three nanolaminate samples and (**b**) their corresponding histogram of thickness of different layers of nanolaminates. The combination of precursors is shown in the legend. A and Z present Al(NO_3_)_3_ and ZrOCl_2_, respectively, and in the figure before A and Z represent the concentration of precursors.

Additionally, it was also demonstrated that the conclusions drawn above were suitable for nanolaminates. First of all, the TEM result showed smooth interfaces of nanolaminates, as shown in Figure 5a. That benefits to the coating of next layer. As meanwhile, the layers were dense and had good contact with each other, which was helpful to avoid defects, such as holes. Secondly, the cross-sectional EDS image of nanolaminates showed that there was no diffusion between Al_2_O_3_ and ZrO_2_, as shown in Figure 5b. Thus, the influence of solution permeation caused by the next coating was avoided, which guaranteed the relative independence of thickness and density. Lastly, as the three samples showed in Figure 6b, the thickness of layers in nanolaminates fabricated by the same precursor were uniform. The ratio of thickness was approximate to the ratio of precursor concentration, and the total thicknesses were all around 70 nm. As the results above show, it was verified that 400 °C was the optimized annealing temperature to fabricate nanolaminates.

### 3.2. Performance of Nanolaminates

#### 3.2.1. Optical Properties

**Figure 7:**
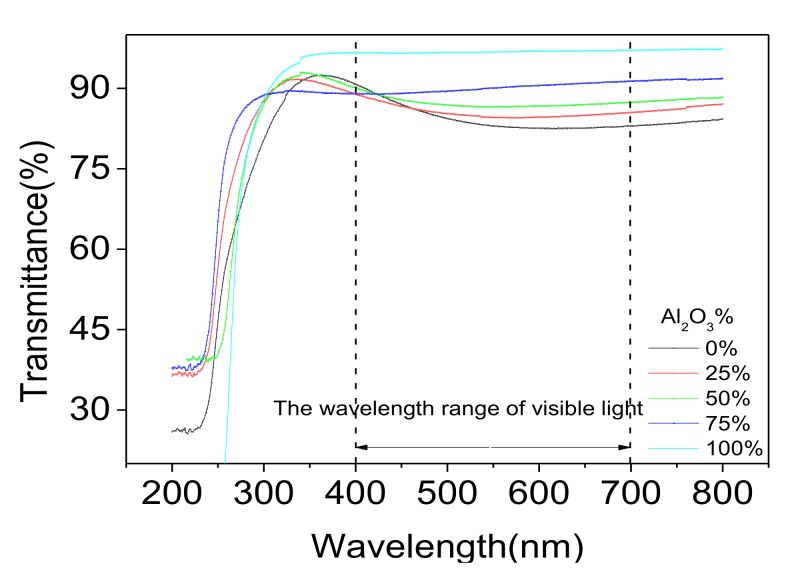
Plot of transmittance versus wavelength.

Transmittance of nanolaminates with different contents of Al_2_O_3_ (as Table 1) were measured, as shown in Figure 7. The transmittance of nanolaminates fabricated by pure ZrO_2_ was around 82%, within the range of visible light. With the increase of Al_2_O_3_ content, the transmittance was obviously improved and it reached the highest value when the dielectric was fabricated with pure Al_2_O_3_.

The optical energy gap of nanolaminates was determined by Tauc formula:

(3) $${\alpha \mathrm{hv}\;=\;A(\mathrm{hv}-E}_{g}{)}^{\frac{1}{2}}$$

where A is a constant, hv and E_g_ are symbols of photon energy and optical energy gap, and α is the optical absorption coefficient [21]. As shown in Table 2, it was found that the optical energy gap of nanolaminates changed from 4.232 eV to 4.89 eV, exactly between the value of ZrO_2_ and Al_2_O_3_ dielectrics. With the content of Al_2_O_3_ increased by 25%, the optical energy gap of nanolaminates were increased around 0.2 eV at the beginning. When the content of Al_2_O_3_ was higher than 75%, it was close to the value of the dielectric made with pure Al_2_O_3_.

#### 3.2.2. Electrical Properties

**Figure 8:**
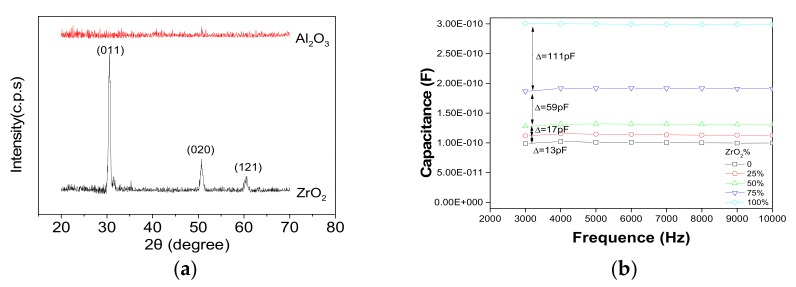
(**a**) X-ray diffraction plot of dielectrics fabricated with pure Al_2_O_3_ and ZrO_2_ (annealing at 400 °C in the air); and (**b**) capacitance versus the frequency plot of nanolaminates with different contents of ZrO_2_. Δ represent the difference value of capacitance.

As shown in Table 3, the dielectric strength of ZrO_2_ was just 0.11 MV/cm. The result measured by XRD showed that the ZrO_2_ has already crystallized and the corresponding crystallization peaks were shown in Figure 8a [22]. That was the main reason why ZrO_2_ was easier to break down. The Al_2_O_3_ was still amorphous while it was poor at capacitance density compared with ZrO_2_. Therefore, the nanolaminate structure was applied to combine the advantages of Al_2_O_3_ and ZrO_2_. The results showed that dielectric strength was improved significantly with the increase of Al_2_O_3_ content. The increment was up to 0.83 MV/cm when the content of Al_2_O_3_ increased from 0% to 25%. Though the variation became smaller when the content of Al_2_O_3_ was higher than 50%, it can also be improved on a small scale, as shown in Table 3. Similarly, the capacitance of nanolaminates grew very slowly at the beginning. However, there was a sudden growth when the content of ZrO_2_ was higher than 50%, as shown in Figure 8b. The capacitance density in Table 3 was the value of capacitance divided by electrode area. Referring to the Formula (2), the relative permittivity was proportional to the capacitance. Therefore, the regular of relative permittivity changed with the content of ZrO_2_ was similar to the capacitance, as shown in Table 3.

The results shown in Table 2 and Table 3 found that the change of the properties was smaller when the content of Al_2_O_3_ was higher than 50%. With the content of Al_2_O_3_ changed from 50% to 100%, the variation of the dielectric strength, capacitance density and relative permittivity was only 0.6 MV/cm, 23.88 nF/cm^2^ and 1.76, respectively. It presented an excellent stability of the dielectric performance. However, the variation of these properties was up to 2.12 MV/cm, 135.35 nF/cm^2^, and 11.64 when the content of Al_2_O_3_ was less than 50%. Thus, there was also a wide scale to adjust the properties of nanolaminates.

#### 3.2.3. The Influence of the Number of Bilayers

Finally, the influence of the number of bilayers in nanolaminates is discussed. As shown in Table 4, the content of Al_2_O_3_ was set to 50% and the number of bilayers was designed as 1, 2, 3, and 6, respectively. The measurement drawn above were carried out. The results showed that the number of bilayers in nanolaminates has little impact on transmittance, optical energy gap, capacitance density, and relative permittivity, as shown in Table 5.

**Figure 9:**
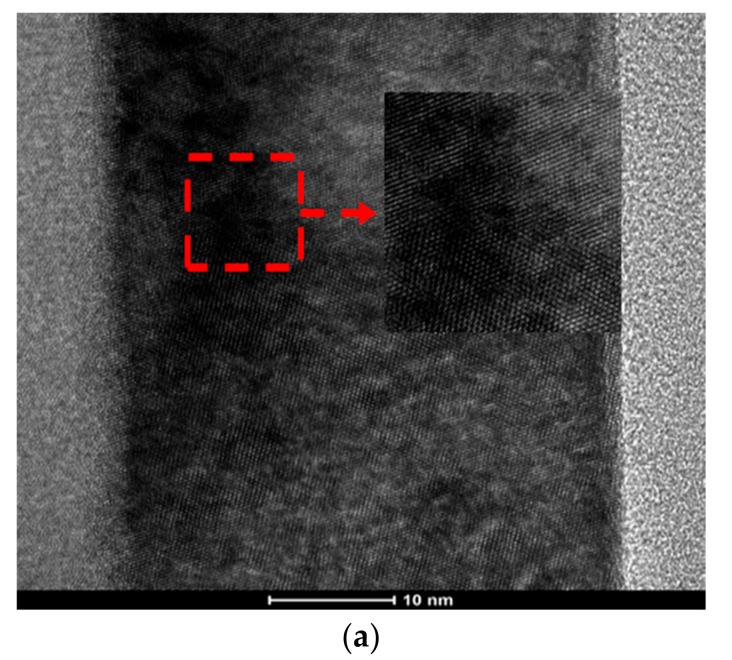

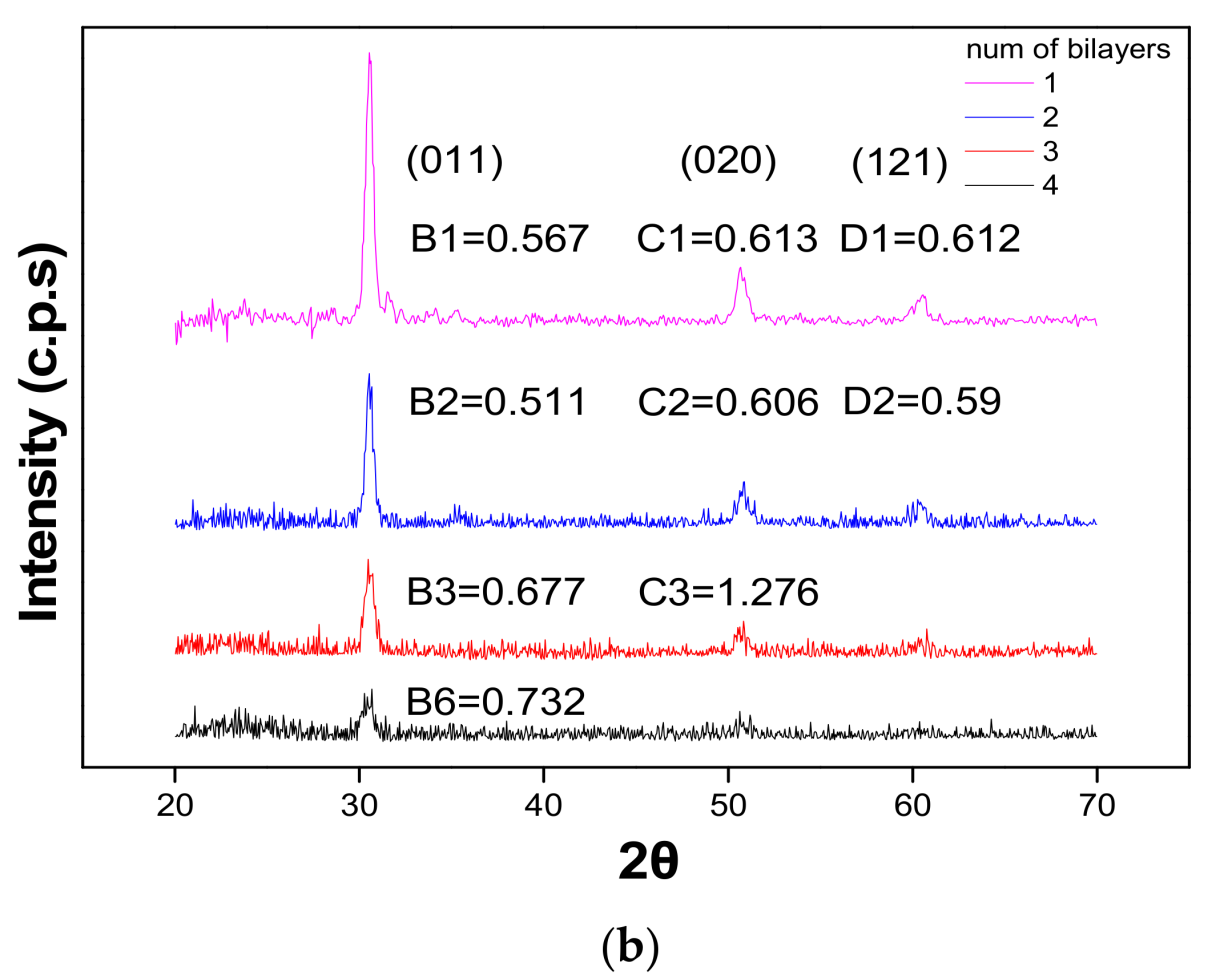
(**a**) HR-TEM image of ZrO_2_; (**b**) X-ray diffraction of nanolaminates fabricated with different numbers of bilayers (annealing at 400 °C in the air). B, C and D are the FWHM corresponding to the (011), (020), and (121) peaks, respectively.

However, the dielectric strength of nanolaminates was influenced significantly by the number of bilayers, as shown in Table 6. The data was analyzed by linear regression and it was found that the dielectric strength was increased by around 0.39 MV/cm with the increase of one bilayer. The general changing tendency of dielectric strength can be concluded as the equation:

(4) $$V_{\mathrm{ds}}\;=\;0.86\;+\;0.39n$$

where n is the number of bilayers. When the bilayer number was up to six, it was comparable to the dielectric fabricated with pure Al_2_O_3_. The main reason was that the crystallite grain boundaries in ZrO_2_ were interrupted by amorphous Al_2_O_3_. Crystallite grain boundaries which contain leakage current spots were located at ZrO_2_ thin films [23,24], as shown in Figure 9a. These boundaries provided paths for leakage current and led to the easy breakdown of the dielectric. However, these paths were interrupted by amorphous Al_2_O_3_ in the nanolaminate structure. It was helpful to improve the dielectric strength. Another reason might be that the variation of crystallite size was not distinct with the change of bilayers. With the increase in the number of bilayers, the thickness per layer was decreased, which might cause a decrease in the average crystallite size. The FWHM corresponding to different peaks were measured by Data Viewer, as shown in Figure 9b, and the average crystallite size was calculated by Scherrer' equation (the average result of the peaks which can be detected by Data Viewer), as shown in Table 6. When the number of bilayers was up to six, the average crystallite size did not present an obvious change. It was helpful to resist the leakage current, which was beneficial to V_ds_ [25,26].

## 4. Conclusions

In summary, 400 °C was demonstrated to be the optimized annealing temperature for nanolaminates fabricated by spin-coating. By changing the content of components and the number of bilayers, the properties control of the dielectric was achieved. Increasing the content of Al_2_O_3_ in nanolaminates was helpful to improve the transmittance, optical energy gap and dielectric strength of dielectric, while the capacitance density and relative permittivity were improved by adjusting the content of ZrO_2_. It was also found that the electrical properties of the dielectric were relatively stable when the content of Al_2_O_3_ was higher than 50% in nanolaminates, while variation of electrical properties became larger when the content of Al_2_O_3_ was less than 50%. Additionally, increasing the number of bilayers in nanolaminates can improve the dielectric strength because the grain boundaries of ZrO_2_ were interrupted by amorphous Al_2_O_3_.

## Figures and Tables

**Table 1:** Nanolaminates with different contents of Al_2_O_3_: n_1_ is the coating time; n_2_ is the number of bilayers in the nanolaminates; and C_1_ and C_2_ are the concentration of Al(NO_3_)_3_ and ZrOCl_2_.

Al_2_O_3_%	n_1_	n_2_	C_1_ (mol/L)	C_2_ (mol/L)
0	2	0	-	0.6
25	6	3	0.1	0.3
50	6	3	0.2	0.2
75	6	3	0.3	0.1
100	2	0	0.6	-

**Table 2:** The optical energy gap (E_g_) of nanolaminates with different contents of Al_2_O_3_.

Al_2_O_3_%	0%	25%	50%	75%	100%
E_g_ (eV)	4.232	4.50	4.66	4.85	4.89

**Table 3:** Dielectric strength (V_ds_), capacitance density (C_d_), and relative permittivity ($\varepsilon_{r}$) of nanolaminates with different contents of Al_2_O_3_.

Al_2_O_3_%	0%	25%	50%	75%	100%
V_ds_ (MV/cm)	0.11	0.94	2.23	2.46	2.83
C_d_ (nF/cm^2^)	238.85	155.25	103.50	91.56	79.62
$\varepsilon_{r}$	20.3	13.2	8.66	7.66	6.9

**Table 4:** Nanolaminates with different numbers of bilayers. n_3_ is the coating time; n_4_ is the number of bilayers in nanolaminates; and C_3_ and C_4_ are the concentration of Al(NO_3_)_3_ and ZrOCl_2_.

Al_2_O_3_%	n_3_	n_4_	C_3_(mol/L)	C_4_(mol/L)
50	2	1	0.6	0.6
50	4	2	0.3	0.3
50	6	3	0.2	0.2
50	12	6	0.1	0.1

**Table 5:** Transmittance (T), optical energy gap (E_g_), capacitance density (C_d_), and relative permittivity ($\varepsilon_{r}$) of nanolaminates with different numbers of bilayers.

T (%)	E_g_ (eV)	C_d_ (nF/cm^2^)	$\varepsilon_{r}$
87 ± 1.4	4.65 ± 0.05	120 ± 7	8.7 ± 0.5

**Table 6:** Dielectric strength (V_ds_) and average crystallite size (D) of nanolaminates with different numbers of bilayers.

Number	1	2	3	6
V_ds_ (MV/cm)	1.09	1.71	2.23	3.14
D(nm)	14.47	15.24	9.42	11.13
